# Simultaneous Determination of Six Isoflavones from *Puerariae Lobatae* Radix by CPE-HPLC and Effect of Puerarin on Tyrosinase Activity

**DOI:** 10.3390/molecules25020344

**Published:** 2020-01-15

**Authors:** Limin Qu, Ke Song, Qi Zhang, Jie Guo, Juan Huang

**Affiliations:** 1 Key Laboratory of Hunan Forest Product and Chemical Industry Engineering, Jishou University, Zhangjiajie 427000, China; liminqu168@163.com (L.Q.); kesong0608@163.com (K.S.); jsuzhangqi@163.com (Q.Z.); 2 Department of Traditional Chinese Medicine, Inner Mongolia Medical University, Inner Mongolia, Huhhot 010010, China

**Keywords:** *Puerariae Lobatae* Radix, cloud point extractions, tyrosinase inhibitors, tyrosinase activator, kinetic analysis

## Abstract

Tyrosinase inhibitors with excellent inhibitory activities and lower side effects have promising applications in the fields of medicine, agriculture, food sciences and cosmetics. In this study, a method for simultaneous separation and determination of six target compounds (puerarin, daidzin, genistein, daidzein, genistin, and formononetin) in *Puerariae Lobatae* Radix was established by cloud point extraction (CPE) and concentration combined with high performance liquid chromatography (HPLC). To achieve high extraction yields, an ultrasound-assisted extraction method was developed based on a salt-modified Triton X-100 system. The optimal extraction conditions are: surfactant Triton X-100 concentration 0.07 g/mL, liquid-solid ratio 80:1 (mL/g), NaCl addition amount 0.6 g, equilibrium time 40 min, equilibrium temperature 70 °C. Under the optimal conditions, the total maximum extraction yield of the six target isoflavones reached 8.92 mg/g. Using l-tyrosine and l-dopa as substrates, the effects of puerarin on the monophenolase and diphenolase activity of tyrosinase activity were investigated by the enzyme kinetics method. The results showed that puerarin inhibited monophenolase activity with an IC_50_ of 0.537 mg/mL and activated diphenolase activity. The inhibition type of puerarin on monophenolase and the activation type of puerarin on diphenolase were analyzed by Lineweaver-Burk plots which show that puerarin showed mixed inhibition on monophenolase and mixed activation on diphenolase. Therefore, puerarin can be used as both a tyrosinase inhibitor and a tyrosinase activator.

## 1. Introduction

Tyrosinase (EC 1.14.18.1), a multifunctional copper-containing enzyme, is widely distributed in plants, animals and microorganisms, and plays an important role in the pathway of melanin biosynthesis from l-tyrosine [1]. It is well-known that tyrosinase can catalyze the first and rate-limiting step of melanin formation, the hydroxylation of l-tyrosine to l-3-(3,4-dihydroxyphenyl) -alanine (l-dopa) (monophenolase activity) and also the subsequent oxidation of dopa to dopaquinone (diphenolase activity). Dopaquinone is highly reactive and can polymerize spontaneously to form melanin in a series of reaction pathways [2,3]. Tyrosinase expression is closely related to many physiological functions in animals. If its function is decreased or deleted, it will lead to depigmentation diseases, such as vitiligo and leukosis [4,5], while autosomal recessive diseases in animals and humans such as Parkinson’s disease are also related to tyrosinase deletion or activity decreases [6,7,8]. In addition, abnormal overexpression of its activity will lead to pigmentation diseases of the human body, such as freckles, chloasma and melanoma [9,10,11,12]. Tyrosinase is also known as a polyphenol oxidase, which is responsible for not only melanization in animals, but also browning in fruits and vegetables. This undesirable enzymatic browning of fruits and vegetables causes a significant decrease in their nutritional and market value [13,14,15]. Therefore, tyrosinase inhibitors with excellent inhibitory activities and lower side effects have promising applications in the fields of medicine, agriculture, food sciences and cosmetics.

Many flavonoids have been identified and exploited as tyrosinase inhibitors in recent years [9]. Within this family is the subclass isoflavones, naturally occurring polyphenolic compounds belonging to the “phytoestrogen” class and exerting pseudo-hormonal activity by binding to estrogen receptors. In addition, isoflavones possess similar activities to those of flavonoids [16]. Isoflavones exist widely in plants from the family Fabaceae, such as *Puerariae Lobatae* Radix (PLR). PLR is the root of *Pueraria lobate* which has been employed as a functional food, as well as an herbal medicine in southern and southeastern Asia for the treatment of fever, diarrhea and diabetes [17]. Moreover, pharmacological studies revealed that PLR exhibits skin-whitening effects for external use and correlational research showed that PLR extract shows tyrosinase inhibition [18].

In recent years, various methods for extracting flavonoids have been developed, such as organic solvent extraction, ultrasound-assisted extraction, microwave extraction, and supercritical fluid extraction [19]. However, some of these methods suffer from the shortcomings of needing specific costly instrumentations, consuming a large amount of organic solvents, low output, or are unsuitable on an industrial scale [20]. To minimize the use of organic solvents and simplify the operating procedure, many other extraction methods have been developed in recent years. Among them, the cloud-point extraction method offers a good and convenient alternative. Nonionic surfactant micelle solutions exist as a single homogeneous isotropic phase at temperatures below their cloud point. However, if the solution temperature is raised above the cloud point, such solutions become turbid and phase separate to yield a surfactant-lean phase (typically dubbed as the aqueous phase) and small volume surfactant-rich (coacervate) phase. Target analyte species often differentially partition between these two phases [21]. Extractions based on such phenomenon are referred to as cloud point extractions (CPEs). Cloud-point extraction has been reported in many studies concerning the extraction and preconcentration of solutes from water [22], urine [23], and soil [24]. However, little was known about the use of surfactant solution as a solvent for the extraction of chemical constituents from herbal products [25,26].

In this study, with the aim of finding the tyrosinase inhibitors, a simple and efficient method based on cloud point extraction and concentration combined with high performance liquid chromatography was developed for the simultaneous separation and determination of six target isoflavones (puerarin, daidzin, daidzein, genistin, genistein and formononetin) in PLR samples. Then we performed tyrosinase inhibitory assays and kinetic analysis to determine the underlying mechanism behind tyrosinase inhibition.

## 2. Results and Discussion 

### 2.1. Optimization of the Cloud-Point System

#### 2.1.1. Effect of the Concentration of Triton X-100

**Figure 1:**
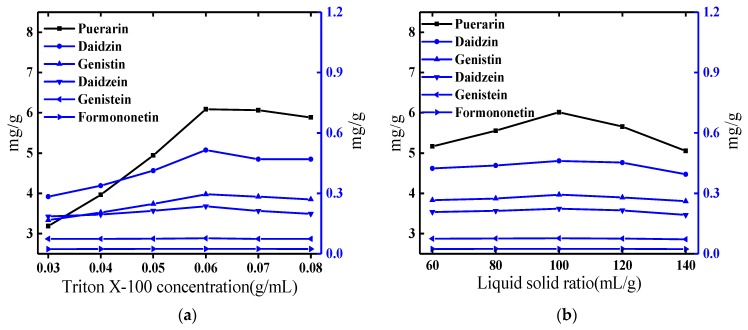

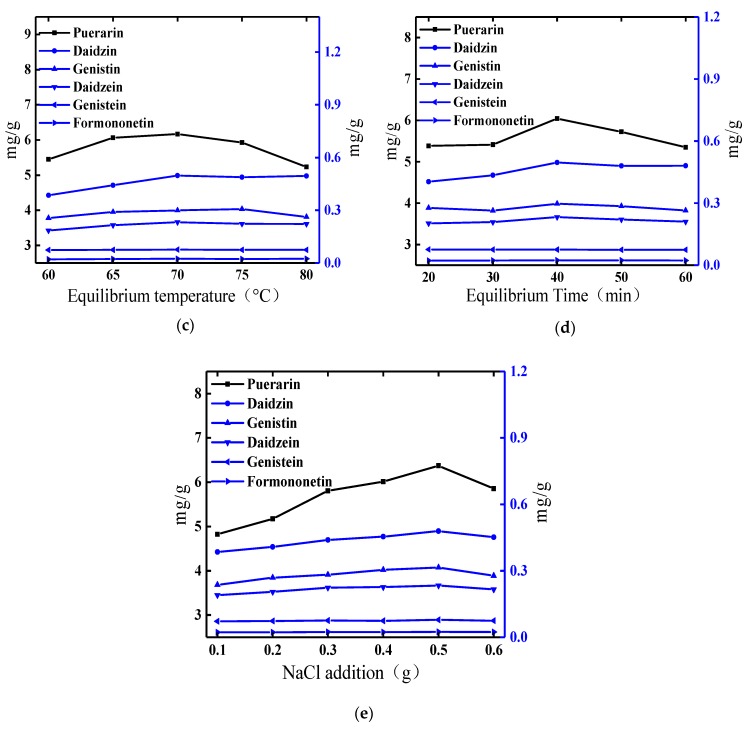
(**a**–**e**) Single factor assay: (**a**) Effect of the concentrations of Triton X-100 on extraction yields of the six target compounds. (**b**) Effect of liquid-solid ratio on extraction yields of the six target compounds. (**c**) Effect of equilibrium temperature on extraction yields of the six target compounds (**d**) Effect of equilibrium time on extraction yields of the six target compounds. (**e**) Effect of NaCl addition on extraction yields of the six target compounds.

The nonionic surfactant Triton X-100 has the advantages of high purity, low toxicity, low cost and the like, and is used as an extractant. For CPE, the extraction yield and the phase volume ratio at the phase separation were influenced by the concentration of the surfactant. Therefore, in this study, the concentration of Triton X-100 in aqueous solution was evaluated in the range of 0.03–0.08 g/mL. As Figure 1a shows, the extraction yield on the relatively hydrophobic isoflavones was significantly influenced by the concentration of Triton X-100. When the Triton X-100 concentration exceeded 0.06 g/mL, the extraction yield decreased slightly. Mass transfer efficiency can be a limiting factor at a higher concentration of a viscous solution. In general, the extension application was limited by the relatively high viscosity of the extraction system because of the slow mass transfer rate. The acoustic cavitation by ultrasound wave could mitigate the effect by higher concentrations and consequently yielded better extraction yields. Based on the experimental results, 0.06 g/mL Triton X-100 was selected for obtaining higher extraction yields.

#### 2.1.2. Effect of Liquid-Solid Ratio

Figure 1b shows the effect of solid–liquid ratio on the six isoflavones’ yields. When the liquid-solid ratio is 100:1 (mL/g), the extracted isoflavones’ contents reached the highest level, and the content decreased with the increase of the liquid-solid ratio, which would due to the increase of the solvent amount, resulting in an increase in the recovery rate.

#### 2.1.3. Effect of Equilibrium Temperature

Equilibrium temperature is an important and efficient parameter in CPE. It is suggested that CPE processes based on the typical temperature-driven phase separation of non-ionic micelle solutions should be performed at temperatures well above the cloud point temperatures of the system. When the equilibrium temperature is higher than the cloud point temperature of the surfactant, the solution will be divided into two phases. Accordingly, the influence of equilibrium temperature within 60–80 °C was studied. The result is shown in Figure 1c, where the extraction effect is best at 70 °C.

#### 2.1.4. Effect of Equilibrium Time

In the CPE method, prolonging the equilibration time facilitates the distribution of the target in the cloud point system, but too long an equilibration time increases the sample processing time. In order to obtain the highest extraction and efficient separation of phases, the equilibration time were optimized. The effect of equilibrium temperature in the range 20 to 60 min was tested. As shown in Figure 1d, when the equilibrium time is 40 min, the extraction effect is the best.

#### 2.1.5. Effect of NaCl Addition

The addition of salt can reduce the cloud point temperature of surfactant, In the CPE method, addition of an electrolyte to a sample solution plays an important role to facilitate the phase separation of the analyte and also increase the transfer of the analyte to surfactant system. In this study, NaCl was selected because of its low cost, commercial availability and non-toxicity. The effect of different NaCl addition was studied. As shown in Figure 1e, when the NaCl addition is 0.5 g, the extraction effect is the best.

### 2.2. Optimization of Orthogonal Experiment Design

In this study, ultrasound-assisted extraction of PLR with a non-ionic surfactant was used to improve the yields of the target compounds. According to the results of single-factor experiments, orthogonal experiments were used to further optimize the relationship between Triton X-100 concentration, liquid-solid ratio, equilibrium time and NaCl addition (Table 1).

**Figure 2:**
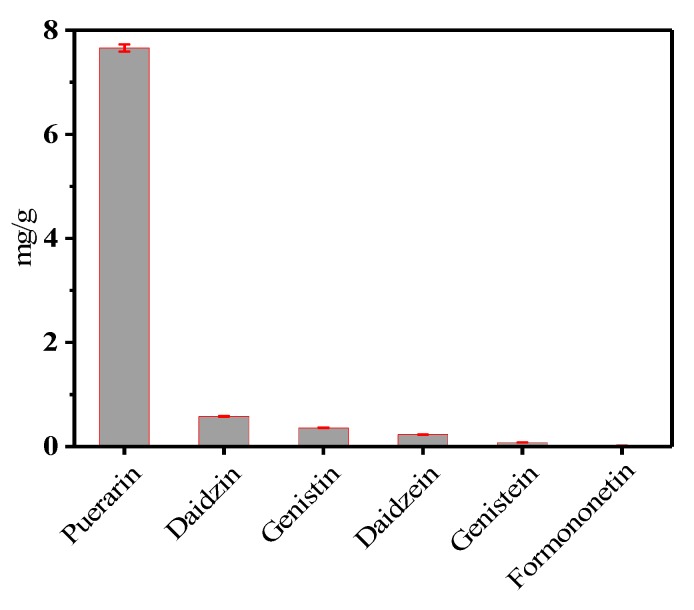
Contents of the six target compounds.

L_9_(3^4^) orthogonal table was used in the experiment. The experimental scheme and results designed by the software SPSS 20.0 are shown in Table 2. The greater the extreme difference of each factor in the visual analysis of orthogonal experiments, the greater the influence of each factor on the experimental results. If the *K* value of a certain level is higher, it indicates that the level is better. According to the visual analysis of the data in Table 2, it can be seen that the primary and secondary factors affecting the total yields of six isoflavones are: Triton X-100 concentration (*A*) > NaCl addition (*C*) > liquid-solid ratio (*B*) > equilibrium time (*D*). The optimal level is *A_3_C_3_B_1_D_2_*, i.e., surfactant concentration is 0.07 g/mL, NaCl addition is 0.6 g, liquid-solid ratio is 80:1, equilibrium time is 40 min, and equilibrium temperature is 70 °C. As shown in Table 3, judging from the *F* value and significance level of each factor, the influence of four factors on the total extraction yield is very significant. The actual total extraction yield of the six compounds was 8.92 mg/g (Figure 2) and the relative standard deviation was 1.36%. These results show that the model is reliable for prediction of expected optimization.

### 2.3. Calibration and Validation of the Analytical Method

**Figure 3:**
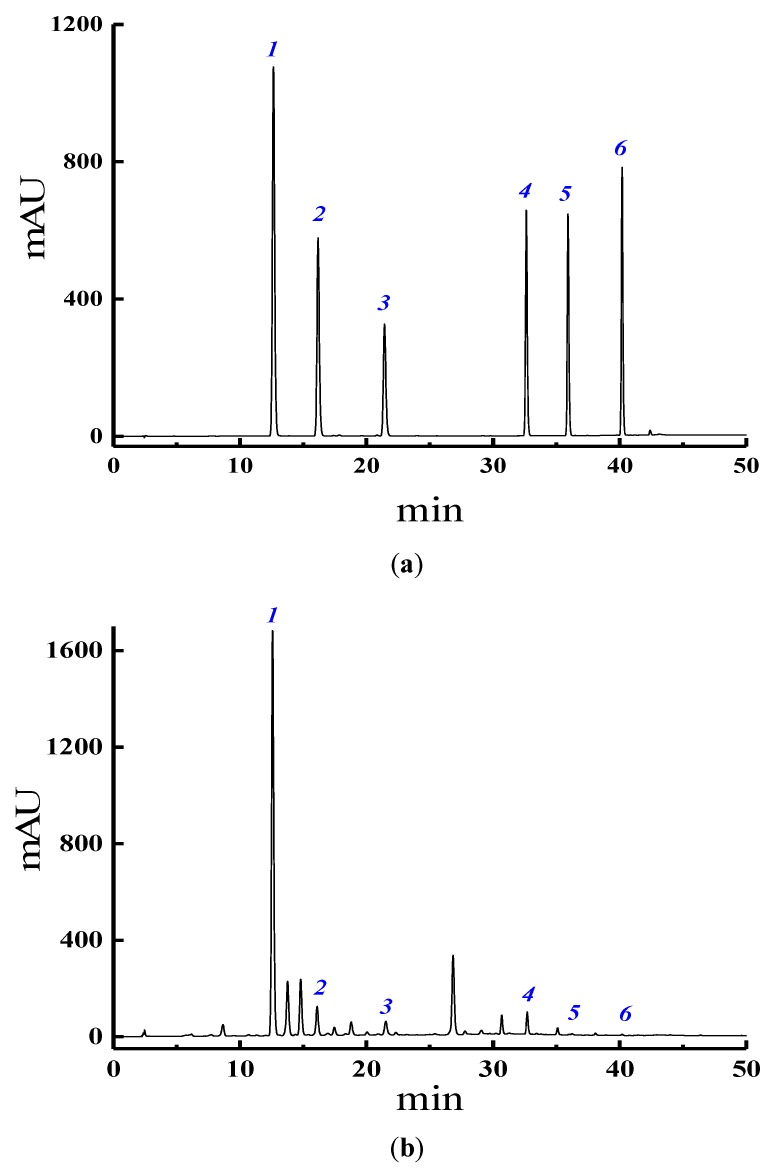
HPLC chromatograms of six isoflavones extracted from PLR, (**a**) standard (**b**) sample. (1: puerarin, 2: daidzin, 3: genistein, 4: daidzein, 5: genistin, 6: formononetin).

HPLC chromatograms of the six isoflavones extracted from PLR is shown in Figure 3. To validate the reliability of the HPLC analysis method, precision (%RSD), stability and the repeatability, as well as recovery were evaluated by the extraction of the analytes from puerarin, daidzin, genistein, daidzein, genistin, and formononetin. The correlation between the concentration of the six isoflavones and the measured value is greater than 0.99. The linear regression equation, correlation coefficient LODs, LOQs and linear range of the six isoflavones are shown in Table S1 in the Supplementary Materials. The precisions, stability, repeatability and recovery of the method were evaluated by six replicate analyses of the spiked samples and recoveries of the method were evaluated by three replicate analyses of the spiked samples. Under optimized conditions, the precisions (RSD) were in the range of 0.40–1.63%, the stability (RSD) were in the range of 1.05–2.41%, the repeatability (RSD) were in the range of 2.52–4.95% and the recovery (RSD) were in the range of 95.12–103.65% for the isoflavones, suggesting that the developed method is reliable. The LODs and LOQs were calculated based on signal-to-noise ratio of 3 and 10, respectively. The LODs and LOQs of the developed method are in the range of 0.0152–0.0307 µg/mL and 0.0506–0.1024 µg/mL, respectively, suggesting that the proposed method is highly sensitive for the determination of puerarin, daidzin, genistein, daidzein, genistin, and formononetin.

### 2.4. Inhibitory Effects of Puerarin on Mushroom Tyrosinase

**Figure 4:**
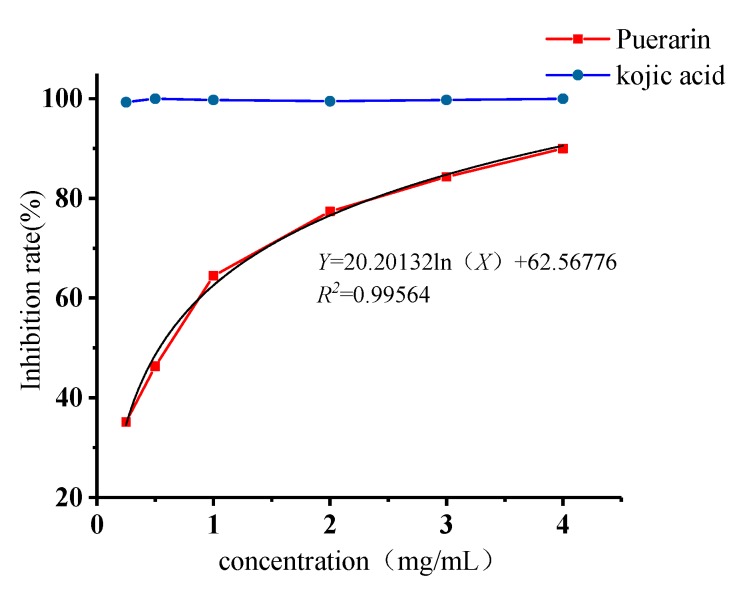
Tyrosinase inhibition rate with l-tyrosine as substrate.

**Figure 5:**
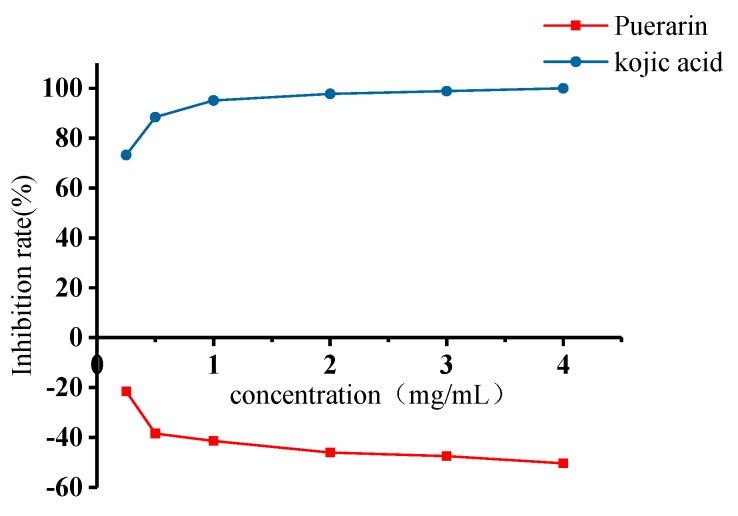
Tyrosinase activation rate with l-dopa as substrate.

The inhibitory effects of puerarin on mushroom tyrosinase were evaluated using kojic acid, the reference material most commonly used to assess tyrosinase inhibitory activity, as positive control, as shown in Figure 4. Inhibitions were measured at a concentration of 0.25, 0.5, 1, 2, 3, 4 mg/mL with the presence 2 mmol/L of l-tyrosine as substrate. With l-tyrosine as the substrate, the inhibition rate of puerarin was positively correlated with the concentration, indicating that puerarin can significantly inhibit the catalytic oxidation process of the enzyme. According to the fitting equation, the IC_50_ value of the semi-inhibitory concentration was 0.537 mg/mL. By changing the substrate and using 2 mmol/L of l-dopa as a substrate, as shown in Figure 5. We found that puerarin can activate the catalytic oxidation process of the enzyme, kojic acid still maintains the activity of inhibiting tyrosinase, and the activation rate is positively correlated with the concentration, probably because of puerarin binding to the group on the enzyme molecule, improving the spatial configuration of the enzyme, so that the enzyme group is better combined with the substrate.

### 2.5. Kinetic Studies of Puerarin Inhibition of Monophenolase Activity

**Figure 6:**
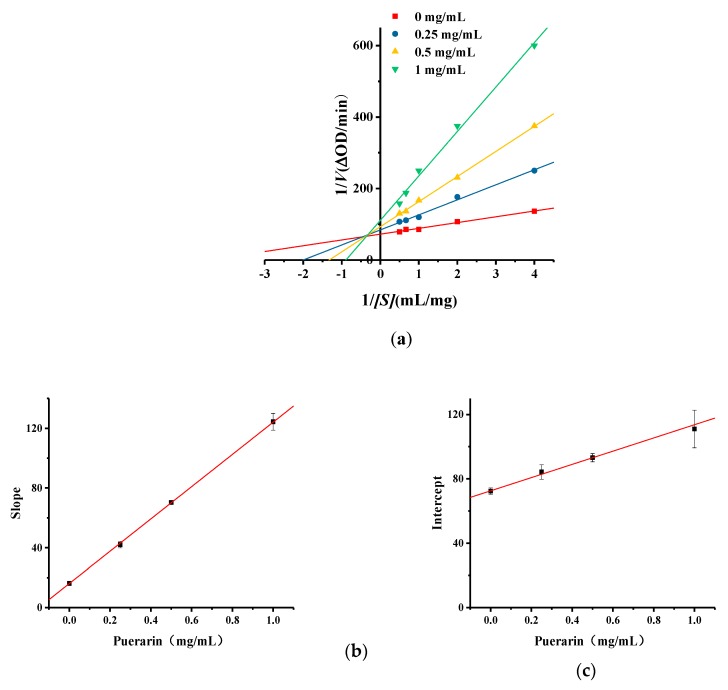
(**a**) Lineweaver-Burk curve of puerarin on tyrosinase monophenolase inhibition; (**b**) The secondary replot represents the slope versus puerarin to determine the inhibition constant *K_i_*; (**c**) The secondary replot represents the intercept versus puerarin to determine the inhibition constant *K_is_*.

Lineweaver–Burk plots were used to investigate the type of inhibition caused by puerarin. As shown in Figure 6a, the images intersect in the second quadrant. The kinetic parameters of puerarin inhibiting tyrosinase monophenolase are shown in Table S2, in the Supplementary Materials. As the concentration of puerarin increases, the *K_m_* value increases gradually and the *V_max_* value decrease gradually, The values of *V_max_* and *K_m_* change evidently with increasing puerarin concentration, indicate that puerarin induced a mixed type of inhibition. That shows puerarin can bind to the enzyme and substrate-enzyme complex simultaneously. Using the quadratic plot of slope and intercept of Michaelis–Menten equation, the inhibition constant *K_i_* of puerarin to free enzyme can be calculated from Figure 6b to be 0.15 mg/mL, and the inhibition constant *K_is_* of puerarin to substrate-enzyme complex can be calculated from Figure 6c to be 1.76 mg/mL.

### 2.6. Kinetic Studies of Puerarin Inhibition of Diphenolase Activity

**Figure 7:**
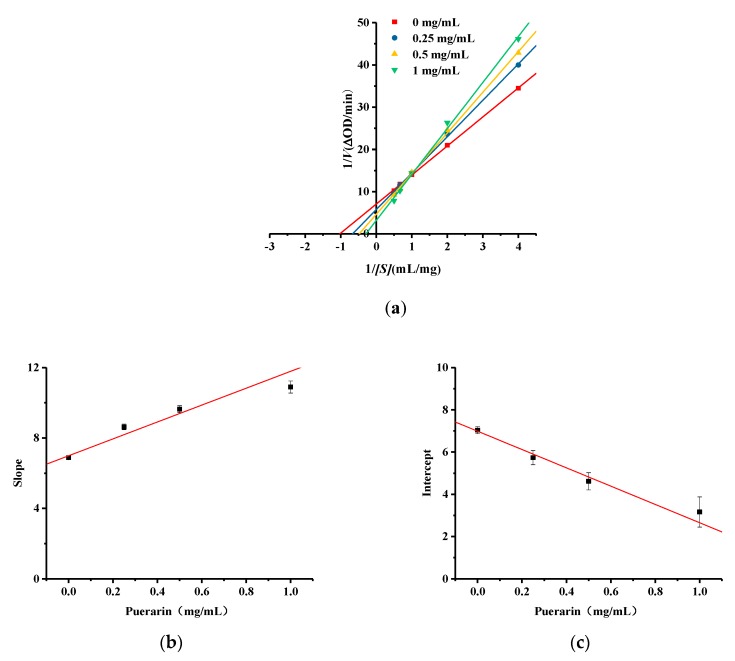
(**a**) Lineweaver-Burk curve of puerarin on tyrosinase diphenolase activation; (**b**) The secondary replot represents the slope versus puerarin to determine the activation constant *K_a_*; (**c**) The secondary replot represents the intercept versus puerarin to determine the activation constant *K_as_*.

Lineweaver–Burk plots were used to investigate the type of activation caused by puerarin. As shown in Figure 7a, the lines intersect in the first quadrant. The kinetic parameters of puerarin activating tyrosinase diphenolase are shown in Table S3, in the Supplementary Materials. As the concentration of puerarin increases, the *K_m_* value increases gradually and the *V_max_* value increases gradually, indicating that for the catalytic oxidation of l-dopa by tyrosinase puerarin displays a mixed type of activation. In the enzymatic reaction system, it could not only affect the combination of substrate and enzyme, but also change the spatial configuration of enzyme and comprehensively improve the reaction speed. Using the quadratic plot of slope and intercept of Michaelis menten equation, the activation constant *K_a_* of puerarin to free enzyme can be calculated from Figure 7b to be 1.45 mg/mL, and the activation constant *K_as_* of puerarin to substrate-enzyme complex can be calculated from Figure 7c to be −1.61 mg/mL.

## 3. Experimental

### 3.1. Materials and Reagents

**Figure 8:**
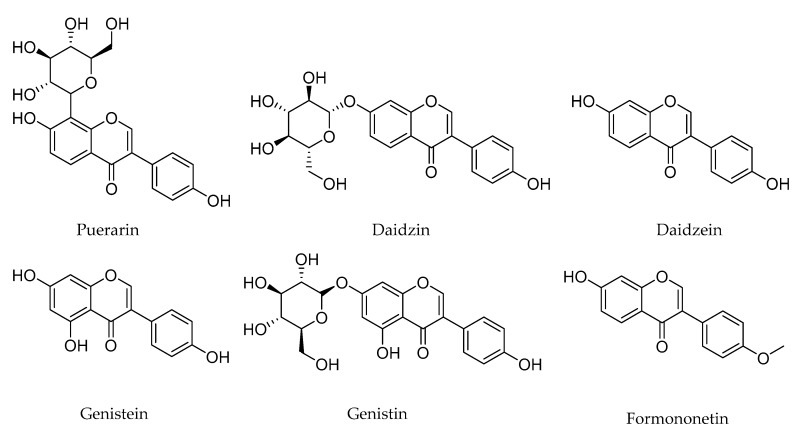
Chemical structures of the six target compounds in PLR.

The PLR samples were harvested from Zhangjiajie City (Hunan, China) in October 2018. The PLR samples were dried at 50 °C in a digital display blower drying oven (ZX-9146-MBE, Shanghai Bosun Industrial Co., Ltd., Shanghai, China) for 24 h, then ground to powder using a crusher (Yb-2000a, Zhejiang Yongkang Yunbang Industry and Trade Co., Ltd., Yongkang, China), sieved (60 mesh) and stored prior to extraction. Triton X-100, Dye xyloside analysis reference substance (HPLC ≥ 98%), l-tyrosine, l-dopa and kojic acid were both purchased from Aladdin Chemicals Co. (Shanghai, China). Tyrosinase (25 KU) was purchased from Sigma Chemicals Co. (Shanghai, China). The standards of puerarin, daidzin, genistein, daidzein, genistin, and formononetin (HPLC ≥ 98%) were purchased from Shanghai Macklin Biochemical Co. (Shanghai, China). Their structures are shown in Figure 8. Other chemical reagents of analytical grade were supplied by Tianjin Concord Technology Co. (Tianjin, China). Deionized water was prepared from an Ultra-Pure Water Meter (Heal Force, Shanghai, China).

### 3.2. Preparation of Cloud-Point System and Extraction Strategy

A salty cloud-point system was developed to extract isoflavones from PLR. Briefly, an accurately weighed amount (0.1 g) of sieved PLR was put into 10 mL centrifuge tube, and extracted with 10 mL aqueous surfactant solution which was obtained at final concentrations of 0.06 g/mL. Then thorough mixing of the PLR and surfactant aqueous solution by a vortex stirrer, the ultrasonic-assisted extraction technique was used to extract isoflavones in PLR for 40 min. The ultrasonic device used (XO-5200DT, 40 KHz, 465 W, Nanjing, China) was equipped with a digital timer and temperature controller. After the extraction step, the aqueous solutions were separated from biomass by centrifugation (at 3500 rpm for 10 min). Transferred the liquid to another 10 mL centrifuge tube, added appropriate amount of NaCl, performed vortex oscillation for 1 min. The two phases can be observed by equilibrating in a constant temperature water bath at 70 °C for 40 min. Centrifugation separated the two phases (at 3000 rpm for 5 min). The upper phase was removed and the surfactant-rich phase was left in the tube and diluted to 2 mL with methanol to reduce the viscosity of the surfactant-rich phase, which was filtered through a 0.45 μm membrane for later use. The quantification of isoflavones in each solution was carried out by HPLC analysis.

### 3.3. Optimization of Orthogonal Experimental Design

The influences of ultrasound-assisted extraction independent variables on the yield of six target compounds were investigated by single factor experiments, the single factor experiment are shown in S1, in the Supplementary Materials. Then, based on these preliminary single factor experiments, four important independent variables were optimized by orthogonal experimental design (OED), including mass concentration of surfactant (*A*), Liquid-solid ratio (*B*), NaCl addition (*C*), Equilibrium time (*D*), twenty-seven experiments with each replicates of the center point were conducted according to a four-factor-three-level L_9_(3^4^) (OED) to optimize the extraction process. The total yields of six target compounds were measured. The experimental scheme was designed by the software SPSS 20.0 (IBM Corporation, Armonk, NY, USA).

### 3.4. HPLC Analysis of Six Target Isoflavones

The quantification of the six target compounds in PLR was performed using a model 1260 reversed-phase HPLC system (Agilent, Waldbronn, Germany). Stock solutions of the six target compounds were prepared by dissolving accurately weighed amounts of puerarin, daidzin, genistein, daidzein, genistin, and formononetin in methanol, diluted with 50% methanol to obtain target compounds stock solutions with final concentrations of 1.0 mg/mL, 5.9 mg/mL, 3.8 mg/mL, 3.7 mg/mL, 3.5 mg/mL and 3.6 mg/mL, respectively. ZORBAX SB C18 reversed-phase column (4.6 mm × 250 mm, 5 μm) was used to analyze the six main isoflavones in PLR. The mobile phase consisted of 0.2% phosphoric acid water (A) and methanol (B). The target compounds were quantified at wavelengths of 250 nm. The flow rate was 1 mL/min, the injection volume was 20 μL, and the column temperature was set at 35 °C. The gradient elution was performed as following: 0–24 min, 20–40% (B); 20–40 min, 40–70% (B); 40–47 min, 70–70% (B); 47–50 min, 70–20% (B).

### 3.5. Calculations of Six Target Isoflavones Extraction Yield

The formula for isoflavones extraction yield in the surfactant phase is as follows:

(1) $$Y=\frac{C\times V}{1000\times M}$$

In the formula: *Y*: Represents extraction yield (mg/g);

*C:* Represents the concentration of the sample in the surfactant phase (μg/mL);

*V*: Represents surfactant phase volume (mL);

*M*: Represents the dosage of *Puerariae Lobatae* (mg).

### 3.6. HPLC Method Validation

The six target compounds were identified by comparing their relative retention times and UV spectra with standard compounds. These compounds were determined using an external standard method. Peak area was used for quantification. Retention data were recorded using the above described chromatographic conditions. Precision, stability and repeatability tests were performed by analyzing the standard solution six times in a row and analyzing the sample solution. The recoveries of the method were evaluated by three replicate analyses of the sample, the HPLC method validations are shown in S2, in the Supplementary Materials.

### 3.7. Measurement of Tyrosinase Activity

#### 3.7.1. Mushroom Tyrosinase Inhibition Assay

As shown in Table 4, PBS buffer solution, substrate and sample solution are sequentially added into 96-well microplates numbered 1, 2, 3 and 4 using 2 mmol/l-tyrosine and 2 mmol/l-dopa as substrates respectively, kojic acid is the positive control, the multifunctional microplate reader is set at 37 °C, the linear vibrating plate pops up for 10 min, tyrosinase solution is sequentially added into each group of wells, quickly put back, the detection wavelength is 475 nm, absorbance value is measured once every 30 s, and detection is performed for 30 min. The total volume of the reaction was 240 microliters. Record data based on absorbance for 30 min. The semi-inhibitory concentration *IC_50_* is defined as the concentration at which the inhibitory effect of the compound on tyrosinase shows a tyrosinase activity inhibitory rate of 50%.

The rate of the inhibition of tyrosinase activity (*I*%) is determined according to the following formula, if the calculation result is negative, the absolute value of the negative number is the activation rate:

(2) $$I\%=\frac{\left(A_{1\;}{-A}_{2}\right)-\left(A_{3}{-A}_{4}\right)}{A_{1}{-A}_{2}}\times 100\%$$

In this formula: *A*_1_: The absorbance measured at 475 nm of the reaction solution with tyrosinase added without sample; *A*_2_: The absorbance measured at 475 nm of the reaction solution with no sample and no tyrosinase added; *A*_3_: The absorbance measured at 475 nm of the reaction solution added with sample and tyrosinase; *A*_4_: The absorbance measured at 475 nm of the reaction solution with the sample added but without tyrosinase.

#### 3.7.2. Kinetics of Puerarin Inhibiting Tyrosinase Monophenolase Catalytic Reaction

To determine the type of *inhibition tyrosinase monophenolase* induced by the compound puerarin, kinetic analysis was performed using mushroom tyrosinase. The effects of puerarin dosage (0, 0.25, 0.5 and 1 mg/mL) on absorbance of the total system when tyrosinase activity is 125 U/mL and l-tyrosine concentrations are 0.25, 0.5, 1, 1.5 and 2 mmol/L. The inhibition type induced by the puerarin was measured by the kinetic analysis. The determination of the inhibition type is calculated by the double reciprocal form of the Michaelis–Menten equation, which is the Lineweaver-Burk equation [27,28,29]. The maximal velocities (*V_max_*) and Michael constants (*K_m_*) for tyrosinase activity were calculated using Lineweaver–Burk plots at five different l-tyrosinase concentrations. All experiments were carried out in triplicate.

Michaelis–Menten equation:

(3) $$V_{0\;}=\frac{V_{max}\left[S\right]}{K_{m}+[S]}$$

The double reciprocal form of the Michaelis–Menten equation is Lineweaver-Burk equation:

(4) $$\frac{1}{V_{0}}=\frac{K_{m}}{V_{max}}\cdot \frac{1}{\left[S\right]}+\frac{1}{V_{max}}$$

#### 3.7.3. Kinetics of Puerarin Activation of the Catalytic Tyrosinase Diphenolase Reaction

To determine the type of activation tyrosinase diphenolase induced by the compound puerarin, kinetic analysis was performed using mushroom tyrosinase. The effects of puerarin dosage (0, 0.25, 0.5 and 1 mg/mL) on the absorbance of the total system when tyrosinase activity is 125 U/mL and l-dopa concentrations are 0.25, 0.5, 1, 1.5 and 2 mmol/L. The suppression type was determined by using the Lineweaver-Burk equation, the double reciprocal form of the Michaelis–Menten equation. The maximal velocities (*V_max_*) and Michael constants (*K_m_*) for tyrosinase activity were calculated using Lineweaver–Burk plots at five different l-dopa concentrations. All experiments were carried out in triplicate.

## 4. Conclusions

This work demonstrated that CPE-HPLC could be effectively applied for the simultaneous extraction and determination of isoflavones in PLR. The cloud point extraction system consists of Triton X-100 and NaCl. The optimized conditions of CPE were identified as a Triton X-100 concentration of 0.07 g/mL the liquid-solid ratio of 35:1, the equilibrium time of 40 min, the NaCl addition of 0.6 g and the equilibrium time of 70 °C. The maximum extraction efficiency reached 8.92 mg/g with 7.66 mg/g puerarin, 0.58 mg/g daidzin, 0.36 mg/g genistin, 0.23 mg/g daidzein, 0.08 mg/g genistein and 0.02 mg/g formononetin, respectively. The results obtained support the sample preparation process in this work, which provides the possibility of extracting and reconcentrating analytes of different polarities in a single process. The proposed one-step method is conducive to large-scale extraction and purification of active ingredients from natural products.

Furthermore, for the first time we found that puerarin exerts a bifunctional regulation on tyrosinase which inhibits monophenolase, while it activates diphenolase. The results of kinetic studies show that puerarin has mixed inhibition on tyrosinase monophenolase and mixed activation on tyrosinase diphenolase. The rate of tyrosinase-catalyzed substrate synthesis of melanin is mainly determined by monophenolase, because monophenolase catalysis time has typical delay characteristics, while diphenolase catalyzes the process without a lag time, so puerarin can activate diphenolase and does not affect its overall inhibitory effect on tyrosinase-catalyzed melanin synthesis, and this is not in contradiction with its whitening effect. Therefore, puerarin has a certain whitening effect due to its strong inhibitory effect of monophenolase. The activation of puerarin on diphenolase can promote the production of melanin when combined with l-dopa, which can be used as a blackening agent in black hair shampoo and even provide reference for clinical treatment of vitiligo.

## Figures and Tables

**Table 1:** Factors and levels of the orthogonal design.

Level	Triton X-100 Concentration (g/mL)	Liquid-Solid Ratio (mL/g)	NaCl Addition (g)	Equilibrium Time (min)
1	0.05	80	0.4	30
2	0.06	100	0.5	40
3	0.07	120	0.6	50

**Table 2:** Design and results of L_9_ (3^4^) orthogonal experiments (n = 3).

Experiment Number	*A*	*B*	*C*	*D*	Total Extraction mg/g
1	2	3	3	1	6.92
2	2	1	2	2	7.77
3	3	3	1	2	7.03
4	3	1	3	3	8.47
5	3	2	2	1	7.60
6	1	1	1	1	5.58
7	2	2	1	3	6.74
8	1	3	2	3	6.29
9	1	2	3	2	6.85
*K1*	18.72	21.82	19.34	20.10	
*K2*	21.44	21.18	21.66	21.65	
*K3*	23.10	20.24	22.24	21.50	
*R*	4.38	1.58	2.90	1.55	

**Table 3:** Variance analysis of orthogonal experiments.

Source of Variation	Sum of Squares	Variance	Mean Square	*F* Value	*p* Value
*A*	9.756	2	4.878	125.146	<0.01
*B*	1.262	2	0.631	16.184	<0.01
*C*	4.712	2	2.356	60.446	<0.01
*D*	1.463	2	0.731	18.760	<0.01
Error	0.702	18			
Total	1351.454	27			

**Table 4:** Composition of various reactant solution.

Name	*A_1_*	*A_2_*	*A_3_*	*A_4_*
PBS/μL	80	160	0	80
Substrate/μL	80	80	80	80
Sample/μL	0	0	80	80
Tyrosinase/μL	80	0	80	0

## Supplementary Materials

S1. Single factor experiment and S2. HPLC Method Validation. Table S1. Linear regression equation, correlation coefficient and linear range of six isoflavones from PLR. Table S2. Kinetic parameters of puerarin inhibiting tyrosinase monophenolase. Table S3. Kinetic parameters of puerarin activating tyrosinase diphenolase. Figure S1. Puerarin on l-tyrosine inhibition process curve. Figure S2. Puerarin on l-dopa activation process curve.
