# Supplementary material for: Simultaneous Determination of Six Isoflavones from Puerariae Lobatae Radix by CPE-HPLC and Effect of Puerarin on Tyrosinase Activity
Source: Molecules. 2020 Jan 15;25(2):344. doi: 10.3390/molecules25020344 (PMC7024166; doi:10.3390/molecules25020344)

molecules

Supplementary Information

Article

Simultaneous determination of six isoflavones from *Puerariae Lobatae* Radix by CPE-HPLC and effect of puerarin on tyrosinase activity

Limin Qu[1], Ke Song[1], Qi Zhang[1], Jie Guo[1,*] and Juan Huang[2,*]

1 Key Laboratory of Hunan Forest Product and Chemical Industry Engineering, Jishou University, Zhangjiajie 427000, China;
2 Department of Traditional Chinese Medicine, Inner Mongolia Medical University, Inner Mongolia, Huhhot 010010, China
* Correspondence: guojie1981@jsu.edu.cn (G. J.); xyxdyd@hotmail.com (H. J.); Tel.: +86-0744-8231-386 (G. J.); +86-0471- 6657-605 (H. J.)

S 1. Single factor experiment

The effects of the Concentrations of Triton X-100, liquid-solid ratio, NaCl Addition, equilibrium temperature and equilibrium time on the content of 6 isoflavones in PLR were studied by single factor average method based on 2.2 steps and fixed ultrasonic time for 40 min.

S 1.1 Effect of the Concentrations of Triton X-100

Under this conditions (0.4 g NaCl, 100:1 liquid-solid ratio, 70 °C equilibrium temperature, 40 min equilibrium time), the effect of Triton X-100 concentration (0.03, 0.04, 0.05, 0.06, 0.07 and 0.08 g/mL) on the extraction yield of six target compounds.

S 1.2 Effect of Liquid-Solid Ratio

Under this conditions (0.06 g/mL Triton X-100, 0.4 g NaCl, 70 °C equilibrium temperature, 40 min equilibrium time), the effect of liquid-solid ratio (60:1, 80:1, 100:1, 120:1 and 140:1 mL/g) on the extraction yield of six target compounds.

S 1.3 *Effect of Equilibrium Temperature*

Under this conditions (0.06 g/mL Triton X-100, 0.4 g NaCl, 100:1 liquid-solid ratio, 40 min equilibrium time), the effect of equilibrium temperatures (60, 65, 70, 75 and 80 °C) on the extraction yield of six target compounds.

S 1.4 *Effect of Equilibrium Time*

Under this conditions (0.06 g/mL Triton X-100, 0.4 g NaCl, 100:1 liquid-solid ratio, 70 °C equilibrium temperature), the effect of equilibrium time (30, 40, 50, 60 and 70 min) on the extraction

yield of six target compounds.

S 1.5 Effect of NaCl Addition

Under this conditions (0.06 g/mL Triton X-100, 100:1 liquid-solid ratio, 70 °C equilibrium temperature, 40 min equilibrium time), the effect of NaCl addition (0.1, 0.2, 0.3, 0.4, 0.5 and 0.6 g) on the extraction yield of six target compounds.

S 2 HPLC Method Validations

S 2.1 Precision test

The precisions of the method were evaluated by 6 replicate analyses (20 mL each time for the same batch of samples) of the mixed standard (puerarin, daidzin, genistein, daidzein, genistin, and formononetin). Under optimized conditions, the precisions (RSD) were 0.55%, 0.56%, 0.40%, 0.54%, 0.68%, and 1.63%, respectively. The results suggest that the instrument has good precision.

S 2.2 Stability test

The stability of the method were evaluated by 6 replicate analyses (20 mL each time for 0.1 g reagent to prepare sample solution) of the sample (puerarin, daidzin, genistein, daidzein, genistin, and formononetin) at 0, 4, 8, 12, 16, 20 h, respectively. Under optimized conditions, the stability (RSD) were 1.05%, 2.04%, 2.15%, 2.02%, 3.84%, and 2.41%. The results suggest that the above six sample in the test solution had good stability within 24 h.

S 2.3 Repeatability test

The repeatability of the method were evaluated by 6 replicate analyses (20 mL each time for 0.1g reagent to prepare sample solution) of the sample (puerarin, daidzin, genistein, daidzein, genistin, and formononetin). Under optimized conditions, the repeatability (RSD) were 3.41%, 2.89%, 3.99%, 2.52%, 4.95%. The results suggest that the above six components in the test solution had good repeatability.

S 2.4 Sample spike recovery test

The recoveries of the method were evaluated by 3 replicate analyses of the sample (puerarin, daidzin, genistein, daidzein, genistin, and formononetin). Under optimized conditions, the average recoveries (RSD) were 99.18%, 101.37%, 95.12%, 95.74%, 96.36%, 103.65%, suggesting that the developed method is recovering rate is high.

Table S1 Linear regression equation, correlation coefficient, LODs, LOQs and linear range of six isoflavones from PLR

Ingredient	Equation of linear regression	R^2	*LODs* (μg/mL)	*LOQs* (μg/mL)	Linear range (μg/mL)
Puerarin	*Y*=66.99174*X*+25.61262	1	0.0239	0.0796	0.8~1000
Daidzin	*Y*=64.45332*X*-33.67189	0.99996	0.0275	0.0917	0.472~590
Genistin	*Y*=60.22372*X*-28.17396	0.99996	0.0307	0.1024	0.304~380
Daidzein	*Y*=87.09195*X*-112.21458	0.99988	0.0177	0.0591	0.296~370
Genistein	*Y*=87.83369*X*-257.76357	0.99944	0.0227	0.0757	0.280~350
Formononetin	*Y*=89.5584*X*-60.89792	0.99992	0.0152	0.0506	0.288~360

Table S2 Kinetic parameters of puerarin inhibiting tyrosinase monophenolase

C((mg/mL))	Michaelis menten equation	K_m(mg/mL)	V_m(ΔOD/min)
0	1/*V*=16.20436/[*S*]+72.30936	0.2241	0.0138
0.25	1/*V*=42.0722/[*S*]+84.22699	0.4995	0.0119
0.5	1/*V*=70.24255/[*S*]+93.11736	0.7543	0.0107
1	1/*V*=124.34473/[*S*]+110.98256	1.1204	0.0090

Table S3 Kinetic parameters of puerarin activating tyrosinase diphenolase

C((mg/mL))	Michaelis menten equation	K_m(mg/mL)	V_m(ΔOD/min)
0	1/*V*= 6.88464/[*S*]+ 7.03802	0.9782	0.1421
0.25	1/*V*= 8.63404/[*S*]+ 5.73753	1.5048	0.1743
0.5	1/*V*= 9.64016/[*S*]+ 4.61532	2.0887	0.2167
1	1/*V*= 10.90067/[*S*]+ 3.16078	3.4487	0.3164

Figure S1 Puerarin on L-tyrosine inhibition process curve

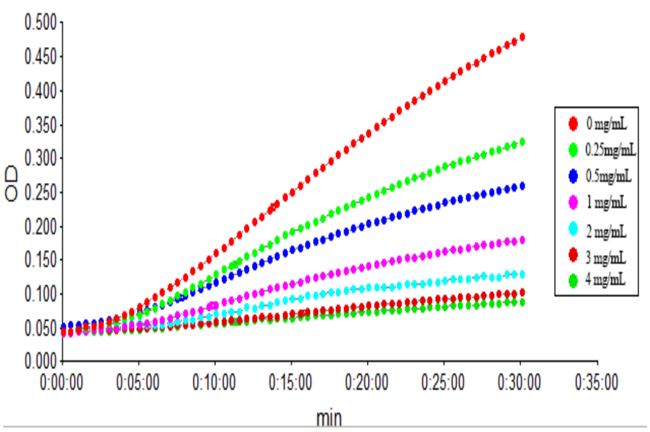

Figure S2 Puerarin on L-dopa activation process curve

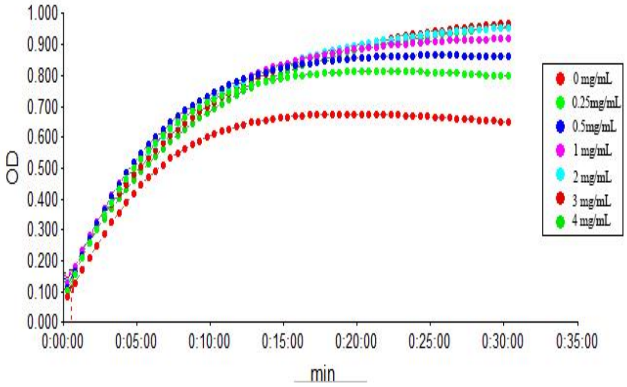

Supplement: Supplementary file 1 [file molecules-25-00344-s001.zip › molecules-693344-supplementary.pdf]
